# Felons

**Published:** 1877-01

**Authors:** 


### FELONS.

Nothing can be more inconvenient, perplexing and painful, than a felon on any one of your ten fingers and thumbs. To have a felon, is to be utterly incapacitated for business or pleasure. You are only calculated to sit in a comfortable armchair, look about you for a more eligible spot, and then frown, scold and grunt. You hold the poor, sick finger up to your gaze and scrutinize it from every point. You feel of it, compare it with its fellow, and not percieving any difference in size or conformation, wonder why in fury it *does* hurt so. You stick it into hot lye, (at a loving neighbor's suggestion) but that doesn't seem to make it pain much worse, so you thrust it into a lemon, and hold that up a while to the admiration of the baby, who stoutly insists to suck it—finger and all. Poultices are next applied, the culprit is carried lovingly upon a pillow, while the owner converts the entire house into a grand promenade ground, in his vainless attempt to distract his attention from his miserable companion. The doctor calls and says it must be opened—laid bare to the bone, in fact. Glory, what a prospect! The confounded finger, throbbing a thousand times worse than though ground between the jaws of a red-hot vice, has wrought you into a state of nervousness that to poke a finger at you makes you squirm and quiver in every muscle, and to have that cold-blooded surgeon try his glittering bistoury upon his thumb nail, and casually remark that you must lay your finger upon the table while he "rips it open"—why, we protest *that* is beyond human endurance. *Rip* it open, mind you;—that word *rip* is so full of significance—it portends of a deliberate puncture, with a sharp knife, over a wretchedly sore and throbbing finger, upon which to lay a downy feather, from the breast of a humming-bird, would cause you to repeat the lord's prayer in seven languages, and that upon tip-toe! But not only a puncture, but a *rip* to follow it! Any wonder you plead for chloroform, local freezing or total destruction? Any wonder, after carrying that finger into every room and closet in the mansion, for three consecutive nights, that upon the fourth, you call for a hypodermic injection of morphine? And, having received it, being transported from unutterable pain and anguish, into blissful ease and repose, has that doctor and the wife of your bosom any right to sit by, and laugh at the remarks you make upon the character, habits and lives of the little green devils that are dancing over your tables and chasing each other up and down the picture cords? Now, have they?

But, seriously, reader, if you ever are so unfortunate as to be invaded by a felon, wait not for the suggestions of friends about boiling lye, lemons, soft-soap and salt, and other remedies to "scatter it." But go straightway to the surgeon and allow him to open it freely, to the bone,—"rip it," in fact, and you will save yourself a world of suffering.

A deep seated felon originates under the covering membrane of the bone, way down, under a firm, and tough tendon. The membrane swells, the parts are unyielding, and long before matter forms, the pain is simply unbearable. If you wait for matter to come to the surface, nine cases in ten, you will have a deformed finger, through loss of the bone, and endanger yourself to the loss of your hand as well, for the matter will burrow under the tendons, in its endeavor to get out, until it reaches the palm of your hand. Free inscision, to the bone, *in the very beginning*, relieves the tension on the bone, opens the way for the single drop of matter that forms, and from the bleeding, relieves the inflammation and pain. *Open it early.* This operation can be rendered almost painless, if you will ask your surgeon to freeze the finger for you before the cutting is performed. This is accomplished by means of the atomizer, from which a fine spray of rhigolene is thrown upon the finger, freezing it to complete numbness, so that it can be whittled as you would a lump of ice, and with as little pain.

